# Social Media Exposure and Left-behind Children’s Tobacco and Alcohol Use: The Roles of Deviant Peer Affiliation and Parent–Child Contact

**DOI:** 10.3390/bs12080275

**Published:** 2022-08-08

**Authors:** Li Wu, Liangshuang Yao, Yuanxiang Guo

**Affiliations:** 1 School of Education, Central China Normal University, Wuhan 430079, China; 2 Central China Normal University Branch, Collaborative Innovation Center of Assessment toward Basic Education Quality, Wuhan 430079, China; 3 School of Psychology, Central China Normal University, Wuhan 430079, China; 4 Key Laboratory of Adolescent Cyberpsychology and Behavior (CCNU), Ministry of Education, Wuhan 430079, China

**Keywords:** social media exposure, tobacco and alcohol use, deviant peer affiliation, parent–child contact, left-behind, China

## Abstract

The public has always been concerned about the problem behaviors of children and teenagers (such as cigarette and alcohol use), especially among disadvantaged groups (e.g., left-behind children in China); in the current information era, left-behind children’s use of social media also has increasingly expanded, which has diverse effects on their adaptation. Accordingly, the present study examined the association between exposure to relevant content on social media and left-behind children’s tobacco and alcohol use, as well as the underlying mechanisms—the mediating effect of deviant peer affiliation and the moderating effect of parent–child contact, the gender differences were also investigated. A sample of 515 Chinese left-behind children (*M*_age_ = 13.39 ± 2.52 years, 45.0% girls) was recruited to complete a set of questionnaires assessing the main variables. The results show that social media exposure was positively associated with tobacco and alcohol use and that deviant peer affiliation significantly mediated this relationship. Furthermore, parent–child interaction attenuated the link between social media exposure and cigarette and alcohol use among left-behind girls, but this moderating effect was not statistically significant among left-behind boys. The moderating role of parent–child contact in the association between deviant peer affiliation and tobacco and alcohol use was insignificant in both boys and girls. These findings may have significance in several ways—theoretically, they not only deepen our understanding of the risk factors and mechanism of tobacco and alcohol use among left-behind children in the current information era and the influences of social media use; practically, they provide direction for the health improvement of left-behind children of different genders.

## 1. Introduction

The term “left-behind children” (LBC) refers to children under the age of 18 who have been left behind at their original residence while one or both parents migrated to other places for employment [1], which is a large group due to a substantial number of rural residents seeking better employment opportunities in the city, accompanying the growing urbanization and economic transformation in China—relevant statistics suggest that the number of rural children left behind exceeds 6.97 million [2]. Due to the disadvantages produced by parental migration (e.g., the lack of parental attention and regulation), left-behind children are more likely to suffer from psychological problems, such as emotional disorders, academic challenges, and problem behaviors [3,4,5,6]. Accordingly, ublic and academic interests have always focused on the development and adaptation of left-behind children.

Two of the most prevalent problem behaviors among children and adolescents are the use of tobacco and alcohol. In particular, individuals are starting to try smoking or drinking at a younger age—some Chinese elementary and middle school students started smoking before the age of nine and drank for the first time before the age of thirteen [7,8]. This can cause great harm to them—they will suffer from mental and physical damage, even develop tobacco and alcohol addiction, and are more likely to be engaged in other substances (marijuana and drugs) during adulthood [9]. Due to their developmental deficiencies, left-behind children usually have a higher prevalence of cigarette and alcohol use and suffer more from the harmful effects [10,11,12]. Consequently, identifying the influencing factors of tobacco and alcohol use among left-behind children may facilitate the development of effective prevention programs and their psychological adaptation. 

Regarding the influencing factors, it is widely established that social media (specifically its relevant contents) is a strong predictive factor for substance abuse among adolescents [13]. In the current information age, social media such as WeChat Moment, Qzone, and Weibo are extremely popular among Chinese children and adolescents—96 percent of individuals aged 10 to 18 have used social media, among whom 46% use it every day and 47% use it for more than one hour per day [14]. Social media provides adolescents with a convenient platform for communicating with their friends and presenting themselves [15]. However, it may bring risks, including exposure to unsuitable content, such as substance abuse, which may result in problematic behaviors [16].

In addition, left-behind children are often provided with mobile phones to easily contact their migrant parents [17], which may boost the use of social media. Meanwhile, because there is no effective regulation or mediation, left-behind youngsters are more susceptible to the negative effects of modern technological applications. In recent years, though the relationship between social media exposure and tobacco and alcohol use has been examined among college students and general children and adolescents [16], it remains unknown whether or not this relationship is established in left-behind children and what the underlying mechanisms are. To address these gaps, this study aimed to examine the association between social media exposure and tobacco and alcohol use, as well as their possible underlying mechanisms, among Chinese left-behind children.

### 1.1. Social Media Exposure and Tobacco and Alcohol Use

Self-presentation is a primary motivation for individuals to use social media, where they can present themselves through various manners and information [18]. Due to the lack of regulation and media filtering, there exists objectionable material on social media [19]. Meanwhile, the majority of content is posted by users (mainly peers), who may have a greater influence than general media [20,21]. It is particularly striking that substance-use-related content, such as text descriptions of drinking experiences, videos demonstrating the performance of smoking or vaping tricks, and personal photographs featuring tobacco and alcohol use, is common in adolescents’ social media [22,23]. Moreover, these references are frequently portrayed in a positive manner, whether in the poster’s depiction or in the comments of other users on the posts [22]. According to cultivation theory, individuals’ perception of the world is often shaped by the media [24]. Repeated exposure to tobacco and alcohol content on social media might lead adolescents to believe that tobacco and alcohol use is common and popular among their real-world friends, and further develop positive attitudes toward smoking and drinking [25], both of which would trigger their intention or initiation of smoking or drinking. 

In addition, empirical research has indicated the correlation between social media exposure and tobacco and alcohol usage. For example, studies showed that exposure to depictions of tobacco use on social media influenced college students and young adults’ smoking intentions [26]; exposure to alcohol-related content on social media significantly predicted subsequent increases in drinking behavior among adolescents [16,27]. On the basis of these theoretical and empirical findings, it was expected that exposure to social media would be positively related to cigarette and alcohol use among Chinese left-behind children (Hypothesis 1).

### 1.2. Deviant Peer Affiliation as a Mediator

The peer group (particularly affiliation with deviant peers) is another significant factor influencing adolescent problem behavior [28,29], among which deviant peer affiliation is a significant factor. It refers to the selective interactions with peers who engage in deviant behavior, such as cheating, violence, and substance abuse, and may pose a significant threat to individuals’ growth and adaptation [30,31]. Specifically, the social learning theory suggests that children and adolescents will imitate deviant behaviors from peers [32,33], and deviant peer affiliation has been identified as a key predictor of adolescents’ tobacco and alcohol use [34,35]; simultaneously, a study found that adolescents with peers engaging in deviant behavior are more likely to engage in substance use [36].

Additionally, other environmental factors, such as media exposure, may also influence deviant peer affiliation. For example, adolescents with greater exposure to smoking in films are more likely to associate with smokers [37]. At the same time, social media depictions of substance use may convey social acceptance and normalcy [38], making adolescents perceive those who smoke and drink to be popular, cool [39], and smart [40]. Moreover, due to the high degree of overlap between adolescents’ social media friends and their offline friends [41], adolescents may also interact with these friends offline on this content. Due to the disadvantages (e.g., lack of parental regulation) caused by parental migration, left-behind children may be more susceptible to media risks and the subsequent negative influences. Furthermore, in the context of the exposure to tobacco and alcohol references on social media, left-behind children are more likely to associate with deviant peers, which, in turn, increases their likelihood of tobacco and alcohol use. We would therefore hypothesize that deviant peer affiliation might mediate the association between social media exposure and tobacco and alcohol use among left-behind children (Hypothesis 2).

### 1.3. The Moderating Role of Materialism

Moreover, family-related issues also greatly influence the development of adolescents (particularly their problem behaviors) [42]. The ecological system and ecological techno-microsystem theories indicate that the family is the most direct and influential micro-system on child development, frequently interacting with other factors such as the media and peers [43]. Moreover, positive family factors may mitigate the adverse effects of other variables [44]. According to the social bonding theory, the bond between children and parents is a significant factor in reducing problem behavior, especially among disadvantaged children [45,46]. For example, parent–child contact in migratory families may be one of the parenting strategies to compensate for the parent–child separation and also a way to maintain the social bonding between parents and left-behind children [47]. In addition, frequent parent–child contact can foster parent–child relationships, providing parental support, warmth, concern, and care for left-behind children [48,49]. All these factors can assist left-behind children in coping with and resisting the influence of perceived negative influences (such as negative content on social media and association with deviant peers), thereby reducing the possibility of deviant behavior.

Regarding the associations among media exposure, peer affiliation, and problem behaviors, although no studies have examined the role of parent–child contact as a moderator, research on positive parenting can provide indirect evidence. As an example, the mother–child relationship has been found to significantly moderate the relationship between exposure to risky environments (such as community violence) and externalized problem behavior among middle school students [50]. Parental concern and warmth, as well as parental control and monitoring, were found to mitigate the negative impact of deviant peer affiliation on adolescents’ problem behaviors [44,51,52]. In addition, it was found that regular parent–adolescent communications are a significant protective factor against left-behind adolescents’ problem behaviors [53]. On the basis of these findings, it was hypothesized that parent–child contact would moderate the associations between deviant peer affiliation and tobacco and alcohol use (Hypothesis 3a), and between social media exposure and tobacco and alcohol use (Hypothesis 3b); specifically, we hypothesized that these associations would be attenuated among left-behind children with more parent–child contact. 

### 1.4. Gender Differences

In addition, significant gender differences may exist in the associations. For example, a relevant study found that the relationship between social media alcohol exposure and drinking was more pronounced among male college freshmen than female freshmen [54]. In contrast to their male counterparts, female adolescents who use social media more are more likely to consume alcohol and engage in binge drinking [55]; however, a longitudinal study indicated that there was no gender difference in the effects of media exposure on alcohol consumption, according to [27]. In addition, research on the gender differences in deviant peer affiliation and its influence reveals that boys are more likely to be engaged in deviant peer affiliation than girls [56] and thus more susceptible to its negative influence [52]. Similarly, there are gender differences in parenting’s protective role. For instance, researchers discovered that parental support and monitoring mitigated the effect of peer group affiliation on alcohol use among girls but had the opposite effect among boys [57]. In general, there exist contradictory findings in the investigation of gender differences that require further examination, and this study, therefore, conducted an exploratory analysis of the potential gender differences.

### 1.5. Current Study

To sum up, this study aimed to comprehensively examine the factors that influence tobacco and alcohol use among left-behind children in China, based on relevant theoretical and empirical evidence. Under the perspective of the theories of cultivation, social learning, and social bonding, the purpose of this study was to define a moderated mediation model to examine the relationship between social media exposure and tobacco and alcohol use among Chinese left-behind children, along with possible internal mechanisms and potential gender differences.

## 2. Materials and Methods

### 2.1. Participants

The convenience sampling method was adopted, and the participants were recruited from two elementary schools (grades 4, 5, and 6), two middle schools (grades 7 and 8), and two high schools (grades 10 and 11) in central China (where there exists the largest number of left-behind children); the left-behind children (whose one or both parents migrate to other places for employment) in these four schools were invited to complete a set of questionnaires voluntarily, with a gift of about CNY 6 as a token of our appreciation. Through this procedure, a total of 549 left-behind children participated in the present study voluntarily. Twenty-eight surveys with incomplete demographic information (age and gender) and six incomplete surveys (more than 30% of the survey items were not answered) were excluded. Finally, 515 left-behind children were included with an average age of 13.39 years (*SD* = 2.52); 283 boys (*M_age_* = 13.49, *SD* = 2.56) and 232 girls (*M_age_* = 13.26, *SD* = 2.47); 185 (35.9%) children were from families of both-parent migration, and 330 (64.1%) children were from families with one-parent migration; the children were left behind for an average of 5.91 years (*SD* = 1.55).

### 2.2. Measurement

#### 2.2.1. Social Media Exposure

Based on the measurement of exposure to alcohol-related SMS content, this study adopted the six items developed by Boyle [54], and individuals were asked how frequently they saw content (including text and images) about drinking or smoking in three types of Chinese typical social media—WeChat Moment, Qzone, and Weibo (e.g., saw drinking-related information on WeChat Moment)—on a 5-point Likert scale ranging from 1 = Never to 5 = Always. In the current study, Cronbach’s alpha for the scale was 0.90 for the entire sample, 0.90 for boys, and 0.90 for girls.

#### 2.2.2. Deviant Peer Affiliation

The Chinese version of the deviant peer affiliation scale [30] was utilized in this study. Peer deviant behaviors included smoking, alcohol consumption, cheating on school tests, stealing or shoplifting, misbehavior, Internet addiction, skipping or cutting school, and physical and verbal aggression. On a 5-point scale ranging from 1 = none to 5 = almost all, participants were asked how many of their friends exhibited each of eight deviant behaviors during the previous year (e.g., “How many of your friends got drunk last year?”), with higher scores indicating greater deviant peer affiliation. In the present study, Cronbach’s alpha for this scale was 0.84 for the total sample, 0.84 for boys, and 0.83 for girls.

#### 2.2.3. Parent–Child Contact

Referring to previous research [58,59], one item (“How often you and your parents contacted on average?”) was adopted to measure how frequently participants contacted their migrated parents, with the response options of *Everyday* (1), *Once or more times a week* (2), *Half a month* (3), *One month* (4), *Almost no contact* (5). For clarity, we inverted the scores when calculating, with higher scores indicating more frequent parent–child contact.

#### 2.2.4. Tobacco and Alcohol Use

The Chinese version of the tobacco and alcohol use scale [60] was adopted. It includes four items, which measure the frequency and amount of tobacco and alcohol consumption. Participants rated these items on a 6-point Likert scale, from 1 = Never to 6 = 20–30 days. The questions were quantity-related, such as “In the last 30 days, how many cigarettes did you smoke per day on the days you smoked?” Participants were asked to respond on a 6-point scale ranging from 1 (Never) to 6 (ten or more cigarettes per day). Responses were averaged, with higher scores indicating greater tobacco and alcohol consumption. In the present study, Cronbach’s alpha for this scale was 0.81 for the entire sample, 0.81 for boys, and 0.70 for girls.

### 2.3. Procedure

The Academic Committee for Scientific Research at the authors’ university approved this study, and all participants signed informed consent forms that outlined the principles governing their voluntary participation (participants were informed that their responses would be anonymous and that they could withdraw this participation at any time). During the evaluation, participants independently provided demographic data and completed the survey instruments; they were encouraged to respond to each item with care. Following the evaluation, questionnaires were collected on the spot, and each participant received a small gift as a reward.

## 3. Results

The gender differences in social media exposure, deviant peer affiliation, parent–child contact, and tobacco and alcohol use were examined first. The results of one-way ANOVA show that girls scored significantly lower than boys on deviant peer affiliation (*F* = 17.71, *p* < 0.001) and tobacco and alcohol use (*F* = 26.29, *p* < 0.001).

Then, the correlation analysis among the variables was conducted, and the descriptive statistics and correlation coefficients are presented in Table 1. As can be seen, social media exposure, deviant peer affiliation, and tobacco and alcohol use were positively correlated with each other in both boys and girls, and parent–child contact was negatively correlated with deviant peer affiliation in girls. Thus, Hypothesis 1, which predicted a positive correlation between social media exposure and tobacco and alcohol use, was supported for both boys and girls. 

In order to test the moderated mediation hypothesis, we used the PROCESS macro for SPSS (Model 15) to examine the mediating effect of deviant peer affiliation and the moderating effect of parent–child contact among boys and girls, respectively; in addition, age and the duration and type (one or both parents migrate) of being left behind were included into the analysis as the control variables. Particularly, we examined the moderating effect of parental contact on the relationship between deviant peer affiliation and tobacco and alcohol use, as well as the relationship between social media exposure and tobacco and alcohol use. As shown in Table 2, after controlling for age and the duration and type of being left behind, social media exposure was positively associated with deviant peer affiliation (*b* = 0.18, *p* < 0.001) and tobacco and alcohol use (*b* = 0.17, *p* < 0.01), and deviant peer affiliation was also positively associated with tobacco and alcohol use (*b* = 0.24, *p* < 0.001). These results indicate that deviant peer affiliation mediated the relationship between social media exposure and tobacco and alcohol use. Therefore, Hypothesis 2 was supported among left-behind boys.

As shown in Table 2, the interactions between deviant peer affiliation and parent–child contact, as well as social media exposure and parent–child contact on tobacco and alcohol use were not significant *(b* = 0.08, *p* = 0.79; *b* = 0.02, *p* = 1.32), suggesting that the association between deviant peer affiliation and tobacco and alcohol use was not moderated by parent–child contact. Thus, Hypotheses 3a and 3b were not supported among left-behind boys.

As shown in Table 3, after controlling for age and the duration and type of being left behind, exposure to social media was positively associated with deviant peer affiliation (*b* = 0.22, *p* < 0.001) and tobacco and alcohol use (*b* = 0.25, *p* < 0.001), and deviant peer affiliation was also positively associated with tobacco and alcohol use (*b* = 0.32, *p* < 0.001). These findings suggest that affiliation with deviant peers mediated the relationship between social media exposure and tobacco and alcohol use. Therefore, Hypothesis 2 was supported among left-behind girls. 

**Figure 1:**
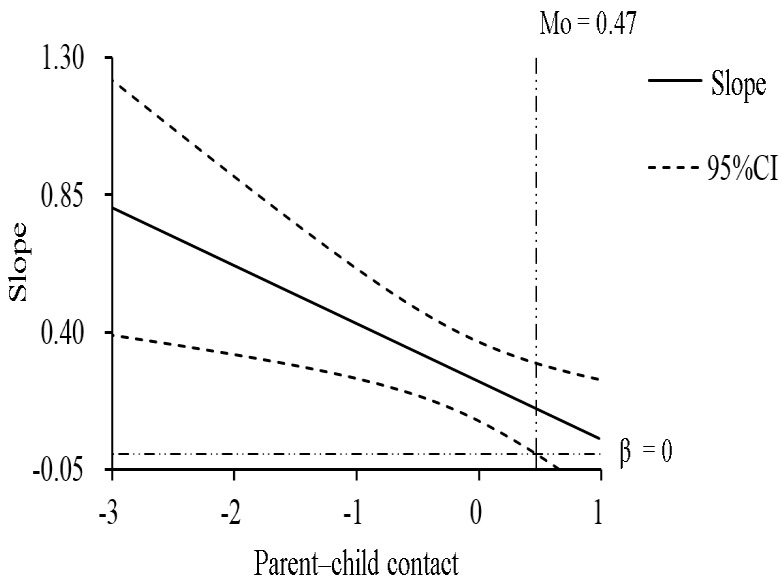
The moderating effect of parent–child contact on the relationship between social media exposure and tobacco and alcohol use among left-behind girls. Note: The horizontal axis is the moderator (parent–child contact), while the vertical axis represents the change in the regression coefficient (i.e., slope) in the regression equation with alcohol and tobacco use as the dependent variable, social media exposure as the independent variable, and parent–child contact as the moderator. All variables were standardized.

In addition, as can be seen in Table 3, the interaction between deviant peer affiliation and parent–child contact on tobacco and alcohol use was not significant (*b* = 0.06, *p* = 0.51), suggesting that the relationship between deviant peer affiliation and tobacco and alcohol use was not moderated by parent–child contact among girls. Thus, Hypothesis 3a was also not supported among left-behind girls. The interaction between social media exposure and parent–child contact on tobacco and alcohol use was significant (*b* = −0.22, *p* < 0.01), indicating that the relationship between social media exposure and tobacco and alcohol use was moderated by parent–child contact. Then, since the moderator (parent–child contact) in this study was a continuous variable, we adopted Johnson–Neyman’s method for simple slope tests. This method is to test simple slopes in the whole range of the moderator and to report the regions in which the simple slopes are significant, overcoming the limitation of the pick-a-point method that can only test simple slopes at several specific levels of the moderators and report whether they are significant or not [61]. As presented in Figure 1, when the parent–child contact (standardized) is within the value range of [−3.03, 0.47], the simple slope is significant; at the same time, the higher the parent–child contact score, the weaker the relationship between social media exposure and tobacco and alcohol use. In other words, the relationship between social media exposure and tobacco and alcohol use among left-behind girls weakened as parent–child contact increased. Thus, Hypothesis 3b was supported among left-behind girls.

## 4. Discussion

It has been well established that the media, peers, and family are significant influences on adolescents’ problem behaviors. This study further examined the effects of social media exposure, deviant peer affiliation, and parent–child contact on left-behind children’s tobacco and alcohol use, as well as the potential gender differences. As hypothesized, exposure to social media was positively associated with tobacco and alcohol use, and deviant peer affiliation mediated this association among both left-behind boys and girls. In the study of left-behind girls, parental contact moderated the relationship between social media exposure and tobacco and alcohol use, but it failed to moderate the relationship between deviant peer affiliation and tobacco and alcohol use; among left-behind boys, no moderating effect could be observed.

### 4.1. Social Media Exposure and Tobacco and Alcohol Use

Consistent with findings from Western studies involving adolescents and college students [20,26], this study further found that social media exposure was directly associated with tobacco and alcohol use among Chinese left-behind children. Researchers believe social media may serve as a “super peer” for adolescents, as it provides models and information about tobacco and alcohol use that significantly affect their behavior [22,62]. Unlike traditional media (such as television and film), social media content is frequently produced by users’ peers [38], and individuals perceived as similar to them are more likely to be imitated [23]. Moreover, through social media exposure, adolescents can continuously get information about their peers’ perceptions, attitudes, and behaviors regarding tobacco and alcohol use [38]. Furthermore, the filtered descriptions of tobacco- and alcohol-related content on social media often romanticize the use of tobacco and alcohol [54]. Thus, adolescents view the use of tobacco and alcohol by peers on social media as positive. In line with the social learning theory [32] and the expectancy–value model [63], those who observe media characters engaging in behaviors such as smoking and drinking without experiencing negative consequences are more likely to adopt these behaviors [54]. Due to the lack of parental care and supervision, left-behind children typically use the Internet and smartphones for recreation and entertainment [64]. They are also more likely to be exposed to harmful tobacco- and alcohol-related content on social media, making them more susceptible to substance abuse. This is also consistent with the cultivation theory, which holds that media-displayed behaviors can influence adolescents to engage in the same actions [24].

### 4.2. Deviant Peer Affiliation as a Mediator 

Deviant peer affiliation was also found to mediate the positive association between social media exposure and tobacco and alcohol use in both left-behind boys and girls. The results are consistent with previous findings that indicate that deviant peer affiliation is a proximal predictor of tobacco use and alcohol use among adolescents and is frequently a mediator between a range of environmental factors and problematic behavior [33]. On the one hand, exposure to tobacco- and alcohol-related content on social media can influence the selection of peers. Social media provides adolescents with extensive social networks for communication. For adolescents whose values must be shaped, peers who present smoking and drinking on social media would be viewed as attractive and even as role models [39,40]. Furthermore, frequent exposure to such unfavorable information may increase adolescents’ tolerance for deviant peer behavior. In other words, adolescents may believe that peer deviant behavior is acceptable and that interacting with them is normal. As left-behind children, they are frequently ignored due to disadvantaged family factors; as a result, they are more likely to participate in online discussions and exchange information they see on social media with their peers in order to express themselves and gain a sense of identity from others [65]. Therefore, frequent exposure to tobacco- and alcohol-related content on social media facilitates the association of left-behind children with peers who use tobacco and alcohol. 

On the other hand, deviant peer affiliation can increase tobacco and alcohol use among adolescents. In accordance with the social learning theory, adolescents not only are able to directly imitate their deviant peers’ problem behaviors, but their peers’ supportive attitudes and acceptable behaviors toward tobacco and alcohol continuously reinforce their smoking and drinking behavior, and they receive social reinforcement for these behaviors [33]. For left-behind children, the influence and significance of peers are amplified. It is important to note that, given the unique circumstances of their parent’s migration, peers are their first confidants and their first source of assistance when they encounter difficulties [66]. Deviant peer affiliation, however, is capable of causing or promoting problematic behavior [67]. Moreover, the media and peers within the ecological techno-microsystem have a synergistic effect on left-behind children, as demonstrated by the mediated effect of the present study. In other words, one ecological risk factor can increase the likelihood that left-behind children will be exposed to another ecological risk, thus increasing the likelihood that they will be affected by multiple risk factors. 

### 4.3. Parent–Child Contact as a Moderator

Intriguingly, parent–child contact moderated the association between social media exposure and tobacco and alcohol use among left-behind girls but not boys. This result demonstrates that the role of parent–child contact in the influence of social media on the socialization of left-behind girls and boys is distinct: it plays a protective role in left-behind girls but has no significant protective role for left-behind boys. Mobile phones and various communication apps, such as WeChat, have made it easier for migrant parents to keep in touch with their children back home. Frequent parent–child interaction can foster an emotional bond between parents and children [49]. Girls not only have greater relational and emotional needs than boys, but they are also more likely to perceive parental care and concern [68]. Therefore, frequent contact with parents can largely satisfy their emotional needs and provide emotional support, thereby protecting them from the detrimental effects of undesirable media content. Meanwhile, boys’ emotional communication with their parents is typically weaker [69]. Without high-quality communication, left-behind boys continue to perceive a weak parent–child bond. Therefore, parental contact cannot shield left-behind boys from the negative influence of risky social media content. In addition, increased parent–child interactions may assist migrant parents in communicating social norms and family education, including media use skills, to their left-behind children and in implementing parental guidance and supervision regarding online media usage. Girls may be more obedient to their parents and more willing to accept parental guidance and supervision; therefore, frequent parent–child contact can mitigate the association between social media exposure and tobacco and alcohol use. However, boys are more likely to perceive parent–child contact as parental monitoring rather than care, and they are also more rebellious and adventurous [70]. Therefore, the protective effect of parental engagement on the relationship between risky content exposure on social media and problem behavior among left-behind boys disappears.

In addition, we discovered that parent–child contact failed to moderate the association between deviant peer affiliation and tobacco and alcohol use among left-behind boys and girls. This result may indicate that, in the absence of parental care and supervision, left-behind children may be subject to a greater and more direct influence from their peers than from their parents. In addition, because peer groups are the primary environmental factors influencing the socialization of left-behind children [71], the influence of these deviant peers cannot be overlooked. Additionally, it may demonstrate that telephonic or video-based communication with left-behind children is insufficient to prevent the negative influences of their peers.

## 5. Implications and Limitations

This research has several limitations. First, the self-reported method used in this study’s measurements may lead to deviations in results; more objective measurement is needed. Second, due to the cross-sectional design, the causal inference could not be established, and relevant studies also suggest that social media use may be more of a symptom than a cause [72]; longitudinal or experimental design should be adopted in future studies, with the aim to uncover the causal link; at the same time, some important factors influencing children’ development and adaptation (such as socioeconomic status) were not included in the current study; other important factors influencing these relationships and the underlying mechanism should be further examined. Finally, though participants were recruited from a single county in central China through convenience sampling, which is a representative sample of Chinese left-behind children, the limited participants may limit the generalizability of the findings, and a broader range of left-behind children can be investigated so as to improve the generality and validity of the findings. 

The findings may also have implications. Theoretically, this study could not only deepen our understanding of the risk factors and mechanism of tobacco and alcohol use among left-behind children in the current information era but also shed more light on the influences of social media use. Practically, these findings may provide direction for the health improvement of left-behind children: first and foremost, it is crucial to monitor their social media usage, especially since they are more susceptible to harmful media content as a result of a lack of parental supervision [64]; in particular, family, school, and society should work together to guide left-behind children to use new media sensibly and correctly. Second, peer interaction should be valued, especially considering the mediating role of deviant peer affiliation, which should be regulated and avoided, and children should be encouraged to interact with good peers. Third, parents should make more contact with their left-behind girls (more phone calls, video calls, etc.). According to the Chinese Left-behind Children Psychological Conditions White Paper [73], high-frequency and high-quality contact between migrant parents and left-behind children is of great importance because the interaction between them is brief (typically during Chinese New Year or the child’s summer break).

## 6. Conclusions

In this study, we identified a positive correlation between social media exposure and tobacco and alcohol use among Chinese left-behind children, as well as the mediating role of deviant peer affiliation in this relationship. The findings indicate that exposure to social media is not only directly related to tobacco and alcohol use among left-behind children but also indirectly through the increase in deviant peer affiliation. The findings also reveal that parental contact could mitigate the association between social media exposure and tobacco and alcohol use among left-behind girls but not boys. Furthermore, the results do not support the notion that parent–child contact moderated the relationship between deviant peer affiliation and tobacco and alcohol use among left-behind boys and girls.

## Figures and Tables

**Table 1:** Descriptive statistics and correlations among study variables.

	Boys	Girls	Correlations			
Variables	*M (SD)*	*M (SD)*	1	2	3	4
1. Social media exposure	1.68 (0.84)	1.55 (0.67)	1	0.28 ***	−0.11	0.30 ***
2. Deviant peer affiliation	1.48 (0.68)	1.26 (0.47)	0.25 ***	1	−0.14 *	0.27 ***
3. Parent–child contact	3.93 (1.01)	4.02 (1.00)	−0.03	−0.08	1	−0.11
4. Tobacco and alcohol use	1.25 (0.62)	1.04 (0.17)	0.27 ***	0.30 ***	−0.04	1

Note: *n_boys_* = 283, *n_girls_* = 232. In the correlation coefficient part, the lower left corner is the data of boys and the upper right corner is the data of girls; * *p* < 0.05; *** *p* < 0.001.

**Table 2:** Testing the moderated mediation effects among left-behind boys.

Regression Equation		Fitting Index		Significance of Coefficients			
Outcome	Predictors	*R^2^*	*F*	*b*	*SE*	LLCI	ULCI
Deviant peer affiliation		0.14	8.86 ***				
	Age			0.11 ***	0.03	0.06	0.16
	Duration of being left behind			0.01	0.04	−0.07	0.09
	Type of being left behind			0.08	0.14	−0.19	0.35
	Social media exposure			0.18 ***	0.06	0.05	0.30
Tobacco and alcohol use		0.18	5.82 ***				
	Age			0.07 *	0.03	0.01	0.12
	Duration of being left behind			0.03	0.04	−0.06	0.11
	Type of being left behind			0.02	0.11	−0.17	0.20
	Social media exposure (SME)			0.17 **	0.07	0.05	0.31
	Deviant peer affiliation (DPA)			0.24 ***	0.07	0.10	0.39
	Parent–child contact (PCC)			0.07	0.06	−0.09	0.20
	SME × PCC			0.08	0.07	−0.06	0.23
	DPA × PCC			0.02	0.06	−0.10	0.13

Note: *N* = 283. Type of being left behind: 0 “both parents migrate”, 1 “one parent migrates”; LLCI = lower limit of the 95% confidence interval, ULCI = upper limit of the 95% confidence interval. * *p* < 0.05; ** *p* < 0.01; *** *p* < 0.001.

**Table 3:** Testing the moderated mediation effects among left-behind girls.

Regression Equation		Fitting Index		Significance of Coefficients			
Outcome	Predictors	*R^2^*	*F*	*b*	*SE*	LLCI	ULCI
Deviant peer affiliation		0.12	5.87 ***				
	Age			0.06 *	0.03	0.01	0.11
	Duration of being left behind			0.01	0.04	−0.08	0.08
	Type of being left behind			−0.08	0.13	−0.37	0.22
	Social media exposure			0.22 ***	0.06	0.09	0.34
Tobacco and alcohol use		0.20	5.51 ***				
	Age			−0.03	0.03	−0.09	0.03
	Duration of being left behind			0.03	0.05	−0.06	0.13
	Type of being left behind			−0.07	0.16	−0.33	0.19
	Social media exposure (SME)			0.25 ***	0.08	0.10	0.41
	Deviant peer affiliation (DPA)			0.32 ***	0.09	0.14	0.50
	Parent–child contact (PCC)			−0.05	0.07	−0.20	0.10
	SME × PCC			−0.22 **	0.08	−0.38	−0.06
	DPA × PCC			0.06	0.06	−0.08	0.13

Note: *N* = 232. Type of being left behind: 0 “both parents migrate”, 1 “one parent migrate”; LLCI = lower limit of the 95% confidence interval, ULCI = upper limit of the 95% confidence interval. * *p* < 0.05; ** *p* < 0.01; *** *p* < 0.001.

## Data Availability

The data of this study are available from the corresponding author upon reasonable request.
